# Meniscus Tear: Pathology, Incidence, and Management

**DOI:** 10.7759/cureus.25121

**Published:** 2022-05-18

**Authors:** Enkhmaa Luvsannyam, Molly S Jain, Ayola R Leitao, Nicolle Maikawa, Ayesha E Leitao

**Affiliations:** 1 Surgery, Avalon University School of Medicine, Willemstad, CUW; 2 Medicine, Saint James School of Medicine, Park Ridge, USA; 3 Internal Medicine, Saint James School of Medicine, Park Ridge, USA; 4 Surgery, Saint James School of Medicine, Park Ridge, USA; 5 Internal Medicine, West Suburban Medical Center, Chicago, USA

**Keywords:** meniscectomy, arthroscopy, repair, degenerative, trauma, meniscus tear

## Abstract

Meniscus tears are a common orthopedic pathology and planning a single, effective treatment is challenging. The diagnosis of meniscal tears requires detailed history-taking, physical examinations, special diagnostic tests, and most likely magnetic resonance imaging (MRI) to confirm the lesion. A good understanding of the meniscal structure including vascularity, zones, function, and affected movements with associated symptoms plays a crucial role in establishing an optimal management plan. A careful assessment of the patient's characteristics, comorbidities, post-repair rehabilitation, and patient’s overall function and satisfaction are also important for ideal management. While conservative management is commonly implemented and the only option for certain patients, partial meniscectomy remains to be the most performed treatment procedure. However, partial meniscectomy is no longer the first-line therapy due to the limitation of certain patient characteristics and side effects in the long run. Instead, meniscal repair has been shown to have better long-term outcomes and is therefore recommended for all tears, especially for young patients with acute traumatic lesions. Tissue engineering has been of high interest in the current research with promising therapeutic results. This review critically evaluates and compares the management of meniscal tears with surgical versus comprehensive management using the current literature.

## Introduction and background

A meniscus tear is one of the most common sports-related injuries and often requires surgery due to pain and dysfunction of the knee [1]. Initially, menisci were described as functionless vestigial remnants and were commonly resected [1]. Increasing scientific research in recent decades discovered the important role of menisci in anatomical and biomechanical functions, including load sharing, stabilizing, shock-absorbing, and lubrication [1]. The incidence of meniscal tears is estimated to be 60 per 100,000 population approximately and the incidence of meniscal-related injuries is rising significantly due to increased sports participation and advanced diagnostic tools [2]. This made meniscus surgery one of the most common orthopedic operations with an incidence of 17 procedures per 100,000 in the United States [2]. Studies found that patients with meniscal injuries have hastened cartilage wear, which predisposes them to early degenerative changes and poor long-term function [2]. In fact, more than 75% of patients with symptomatic osteoarthritis have known meniscal injuries [2].

The treatment options for meniscal tear include nonoperative management, meniscal repair, or meniscectomy [2]. Surgical management is the mainstay treatment of most meniscal tears. The first open surgical repair of a meniscal tear was performed by Annandale in 1885 followed by the development of various arthroscopic techniques [2]. Total meniscectomy was the gold standard management of meniscal tears until the 1970s due to the lack of understanding of the vital role of menisci [2]. However, it became prominent that the patients who underwent meniscectomy developed femoral condylar flattening and joint space narrowing, leading to degeneration [3]. Since then, studies confirmed that the meniscus is an important weight-bearing structure and its absence leads to knee instability and osteoarthritis [1-3]. Therefore, meniscal injury repair and preservation have become a big part of orthopedic research and have advanced significantly only in the last few decades [1-3]. A study by Abrams GD et al. found that the number of meniscal preservation procedures has doubled in the last five years [4]. Changing meniscal treatment from resection to preservation, either surgical or conservative, has shown promising results with shorter recovery times and functional outcomes [2].

Arthroscopic partial meniscectomy (APM) has been the gold standard treatment for meniscal injuries in the last few years around the globe [2,5]. More than 350,000 APMs have been performed from 2005 to 2011 in the United States [5]. However, recent studies suggest that the outcomes of APM are not significantly different from the outcomes of placebo surgery, and the risk of undergoing total knee replacement is three times higher among patients with previous APM [2,5]. A meniscal repair is an effective alternative option to heal meniscal lesions without the adverse effects of partial and total meniscectomy [2]. Although the short-term outcome of meniscus repair shows less than a 10% failure rate at a two-year follow-up, the long-term failure rates have stayed consistent between 23% and 30% despite using various techniques [2]. Nonetheless, meniscal repair still is the preferred method with lower rates of radiographic degenerative changes compared to meniscectomies [2]. Conservative management can be the preferred option for certain patients with smaller tears, advanced osteoarthritis, and those who are unable to undergo operations. A number of clinical trials have concluded that the surgery is not superior to conservative management in degenerative meniscal lesions [5]. Over the years, clinical studies demonstrated that patients with knee osteoarthritis who underwent arthroscopic treatment showed more symptomatic relapse and lower satisfaction rates compared to patients with traumatic meniscal lesions [5]. Choosing the optimal management for meniscal tear remains controversial due to a lack of evidence directly comparing these treatment options. However, it requires a thorough assessment with several factors, including patient age, comorbidities, characteristics of the tear, and symptoms, to establish the appropriate treatment.

This paper aims to review the current literature about the meniscus anatomy, pathology, incidence, and management options of meniscal tears, specifically to compare surgery to conservative management.

## Review

Anatomy of the meniscus

In the knee, menisci are wedges of fibrocartilage that are located between the tibial plateau and femoral condyle [6]. The most plentiful part of the menisci is collagen (75%), predominantly type I collagen (>90%), despite the fact that it additionally contains types II, III, V, and VI [6]. Collagen strands are organized for the most part along a longitudinal or circumferential bearing. The microanatomy of the meniscus is composed of thick fibrocartilage, which is called fibrochondrocyte, as it is a combination of fibroblasts and chondrocytes [7]. These cells are liable for the blend and support of the extracellular fibrocartilaginous matrix. The extracellular network additionally incorporates proteoglycans, glycoproteins, and elastin [7].

**Figure 1:**
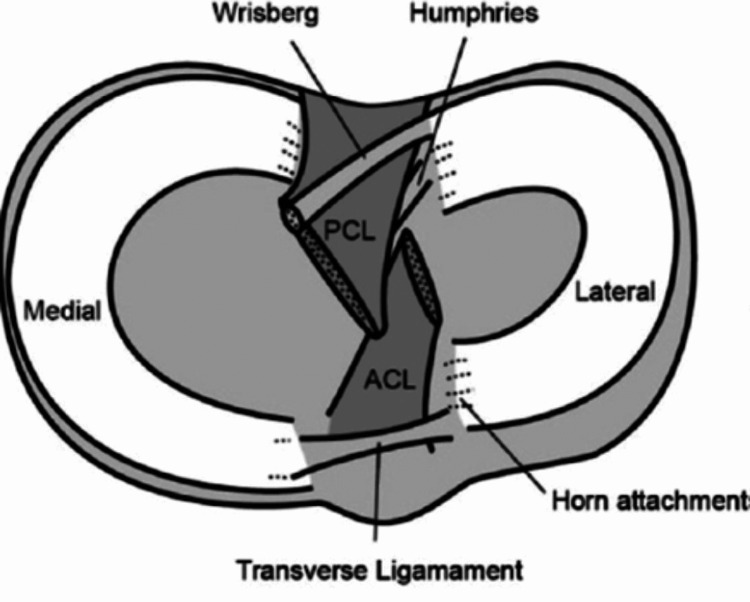
Menisci anatomy viewed in situ on the tibia Note. Image from Bryceland JK et al. (2017) [6]

The larger, semilunar medial meniscus is firmly attached in comparison to the loosely attached, rounder lateral meniscus [8]. Both the anterior and posterior horns of the menisci attach to the tibial plateau. Anteriorly, a transverse ligament connects the menisci; posteriorly, the meniscofemoral ligament holds the posterior horn of the lateral meniscus to the femoral condyle [8]. The peripheral meniscus is connected to the tibia by the coronary ligaments. Although the lateral collateral ligament passes closer to the lateral meniscus, it is not attached to it (Figure 1) [6].

The joint capsule attaches to the entire periphery of each meniscus but is more firmly attached to the medial meniscus [6]. Popliteal hiatus is the area between the joint capsule and lateral meniscus that allows the popliteal tendon to pass through. Contraction by the popliteus during knee flexion pulls the lateral meniscus posteriorly, staying away from entanglement inside the joint space [7]. The medial meniscus does not have a direct solid association. The medial meniscus might move a couple of millimeters while the less steady lateral meniscus might move somewhere around 1 cm [6].

The vascular supply of the knee joint is regulated by the parameniscal capillary plexus whereby the lateral and medial geniculate arteries anastomose [6]. There are three distinct zones of the meniscus, distinguished based on blood supply: the peripheral vascularized red-red zone (zone 1), the avascular white-white zone (zone 3), and the transition between two called the red-white zone (zone 2) [7]. Healing and repair of tissue are directly related to vascularization with blood supply to the tissue, hence making the white-white zone prone to degenerative lesions [7].

Biomechanics and pathology of the meniscus

The menisci are responsible for the majority of load transmission across the knee. Some other crucial functions include increasing joint conformity for maintaining fluid lubrication with synovial fluids [2]. This further maintains congruity between femoral and tibial condyles to assimilate their proper usage. Moreover, energy dissipation, or shock absorption, by the menisci is primarily important when it comes to trauma and high impact load over knee joints [9]. The knee extension and flexion biomechanics are directly related to the motion of the femoral condyle. During extension, the menisci are displaced anteroposteriorly due to force exerted by the femoral condyle [9]. During flexion, the menisci deform mediolaterally, maintaining joint congruity and maximal contact area [9]. The femur externally rotates on the tibia, and the medial meniscus is pulled forward.

**Figure 2:**
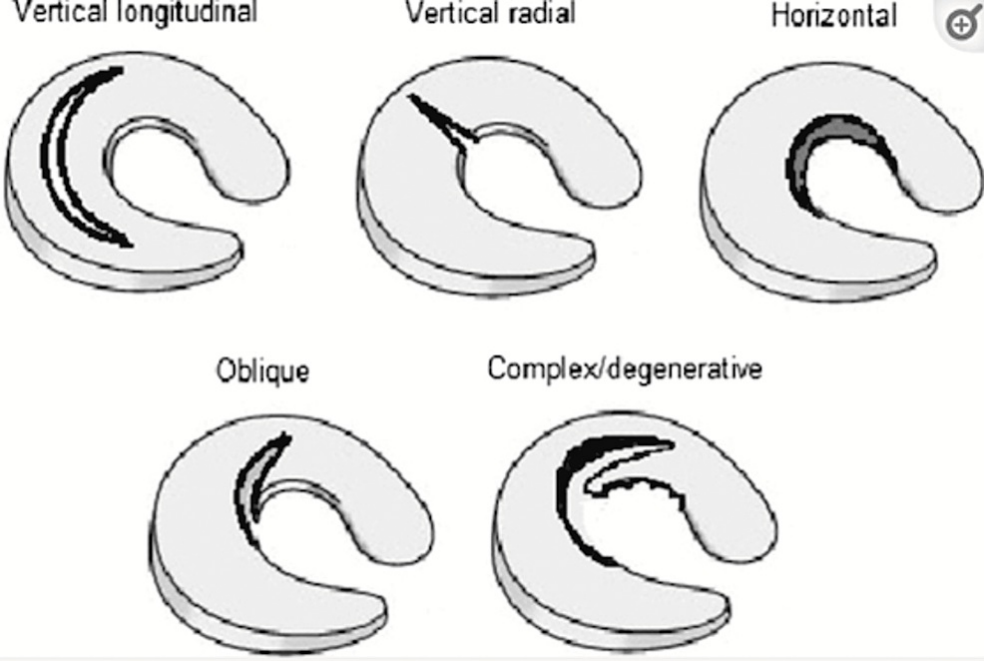
Different types of meniscus tears Note. Image from Karia M et al. (2019) [1]

Meniscus tears can be divided into vertical, horizontal, and complex. Vertical lesions are asymptomatic often and could lead to longitudinal lesions in the periphery of the meniscus [2]. Horizontal tears could be more fatal, leading to complete cleavage between the meniscal edge layers. Complex lesions are related to degenerative changes in the knee, consisting of both vertical and horizontal lesions (Figure 2) [1].

Epidemiology

All populations are subjected to meniscal tears when their knee is subjected to an external force that causes the knee to twist. The prevalence of meniscal tears is approximately 12% to 14%, with an approximate incidence of 61 cases in every 100000 people [10]. Acute trauma-related tears are more prevalent in active young populations and those engaging in sports activities. On the other hand, degenerative meniscus tears affect the elderly population, with the peak onset age in men being 41 to 50 years, while in females, it is 61 to 70. The approximate number of cases per year is 850000. The associated orthopedic surgeries to correct the meniscus tears are between 10% and 20% [10].

Several factors are risk factors for meniscal tears, increasing the likelihood of developing meniscal tears. The non-modifiable risk factors for meniscal tears include sex, where the incidence in men is 2.5 times more than in women [11]. Meniscal tears are more in individuals with a biconcave tibial plateau, a discoid meniscus, those with lower extremity alignment, and those with ligamentous laxity. The modifiable risk factors that increase the risks of developing meniscal tears are a high body mass index, certain occupations, such as squatting, lifting and carrying weights, stairs climbing, and athletes, and those engaging in sports-related activities, including footballers, and those playing rugby.

Several conditions are associated with meniscus tears. Patients with anterior cruciate ligament (ACL) injuries have increased incidences of having meniscal tears with an approximation of 22% to 86% [10]. Acute ACL injury was mainly associated with lateral meniscal tears while chronic ACL injury was associated with medial meniscal tears. According to Valdez et al., although posterior cruciate ligament (PCL) incidence is lower than ACL, approximately 8% of PCL patients develop meniscal tears. Grade III PCL increases the prevalence of meniscus tears [10].

Diagnostics

**Figure 3:**
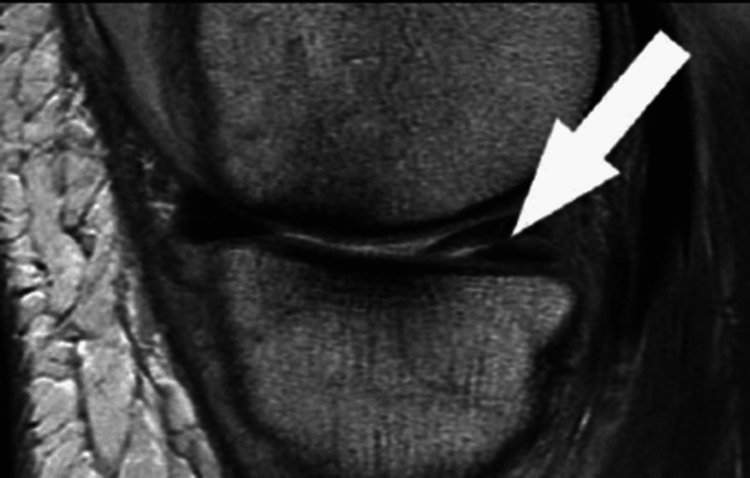
Sagittal MRI of posterior horn medial meniscus horizontal tear Note. Image from Bhan K (2020) [2] MRI: magnetic resonance imaging

A diagnosis of meniscus tears involves a detailed history, physical examination, imaging, and special tests. History-taking information about the cause and presentation of the meniscus tears can be identified. A physical examination can diagnose a torn meniscus. The cause of the signs and symptoms of meniscus tears can be identified when the physicians move the affected lower limbs in different positions, watching the patient walk and squat [2]. Radiological studies are used to generate images to confirm meniscus tears. As the meniscus consists of cartilage, X-rays cannot be used to show a meniscus tear. However, X-ray images rule out other causes associated with symptoms similar to those with meniscus tears. The most common radiographic test used to diagnose meniscus tears is magnetic resonance imaging (MRI). In detecting a torn meniscus, MRI has a specificity of 88% and a sensitivity of 93% [2]. On MRI, the abnormal shape of the meniscus and high signal intensity contacting the surface edge can be appreciated (Figure 3) [2].

McMurray’s test is one of the clinical tests used to diagnose meniscal tears. It is a procedure that involves systemic rotation of the knee by the physician. The test is positive when there is clicking/popping and pain in the knee when the knee is rotated [12]. Another clinical test is Apley’s grinding test where the patient lies in a prone position with flexion of the knees to 90 degrees. This is followed by the physician's medial and lateral rotation of the knee followed by distraction. Compression, instead of a distraction, follows a repetition of the process. A meniscus tear is diagnosed when a decreased rotation is associated with a more painful knee when the knee is rotated and compressed [13].

Treatment

The fibrocartilaginous meniscus is essential for the musculoskeletal stability of the knee joint [14]. Damage or loss of this vital structure can lead to significant articulatory morbidity and an accelerated course of osteoarthritis. Therefore, attempts should be made to preserve the meniscus [14]. Treatment and management of meniscal tears are dictated by multiple factors and include age, the complexity of the tear, tissue quality, the severity of symptoms, etiology (traumatic versus atraumatic tear), and quantified surgical risk [14]. For acutely painful and swollen knees with a suspected meniscus tear, the initial strategy is to follow the R.I.C.E. (rest, ice, compression, elevation) principle [7]. Oral medication, such as acetaminophen and nonsteroidal anti-inflammatory drugs (NSAIDs), can also be prescribed to alleviate pain and swelling [7]. For degenerative tears and simple traumatic meniscal tears, additional conservative management involves the use of a knee brace, activity modification, physical therapy, and quadriceps strengthening exercises. Physical therapy should be initiated early and should begin with pain-free range of motion exercises with progression to weight-bearing exercises as tolerated [7]. Endurance activities like biking and swimming that decrease mechanical load across the knee joint should also be encouraged [7]. In patients who decline surgery or in whom chronic NSAID use/surgery is contraindicated, intra-articular cortisone or hyaluronic injections can also be provided intermittently, every two to three months, for short-term relief [15].

For simple traumatic/degenerative tears, it is reasonable to continue conservative management for about four to six weeks [7]. A study found that quadriceps-strengthening exercises three times a week for 10 weeks have improved knee function by 35% in patients with osteoarthritis [16]. Another randomized controlled trial compared APM followed by supervised exercise and exercise therapy alone in patients with degenerative meniscus tear [17]. They found significant improvement in both groups after eight weeks but no significant difference in outcomes between the groups [17]. Therefore, the author suggested that conservative treatment with supervised exercise should be the first-line management [17]. However, if mechanical symptoms persist, are disabling, and significantly affect the quality of life, surgical intervention should be considered [7]. Generally, surgery is favored if the case scenario involves any of the following: (1) red zone tear, (2) complex and extensive meniscal rips > 1 cm, (3) young healthy candidates with age<40 years old, (4) acute tears that occurred <6 weeks, and (5) the presence of a concurrent ACL injury [14]. The current surgical approach to managing meniscal tear encompasses meniscectomy, meniscal repair, and meniscal reconstruction [14].

Meniscectomy

A meniscectomy (or meniscal resection) can be done completely or partially via an open or arthroscopic approach [14]. In the current era, total meniscectomy is almost never performed due to well-established side effects, most importantly early-onset osteoarthritis [14]. An APM is more commonly performed because it is minimally invasive, associated with shorter recovery time, and lower morbidity comparatively [14]. Indications for APM include radial white-white zone (non-perfused) meniscus tears and degenerative meniscus injuries, which are unresponsive to conservative management [15]. Nevertheless, osteoarthritis still occurs in the long run. Extensive clinical research shows no significant long-term benefits of APM over non-operative management of traumatic as well as atraumatic meniscus tears [15]. Factors associated with poor outcomes of APM included obesity, female gender, and advanced osteoarthritis [15]. Therefore, per current guidelines, APM is no longer the first-line therapy and should be undertaken only in selective patients with non-repairable meniscal tears and those with persistent mechanical symptoms beyond 3 months [14].

Meniscal Repair

A meniscal repair like meniscectomy can also be performed via an open surgical or arthroscopic approach [15]. Arthroscopic repair predominates over open repair due to a lower risk of neural damage. Tear patterns and adequacy of vascularity should be accessed before proceeding with meniscal repair [15]. Repair is most advantageous in the setting of acute traumatic meniscal tears within the well-perfused, peripheral red-red zones of the meniscus. Furthermore, longitudinal/horizontal and vertical tears are more amenable to repair compared to radial tears [14]. However, a repair can still be attempted with radial tears in partially perfused red-white zones [14]. Arthroscopic meniscus repair can be achieved via inside-out, outside-in, and all-inside techniques [14].

The inside-out approach is associated with the greatest success rates and is the golden standard in meniscal repair [14]. In this approach, the sutures are passed from inside the knee to an extra-capsular area through the extra-articular incision and a knot is then secured over the joint capsule [14]. The inside-out technique is commonly used for posterior horn meniscal damage [14]. The outside-in technique is more commonly used for anterior horn tears. In this approach, the spinal needle is passed through the meniscal rip in an outside-in manner [15]. The suture is passed through the arthroscopic portal once the tip of the needle is visible. The suture is pulled back after an interference knot is tied at the end. The operation is continued until the tear is stabilized [15]. The all-inside technique is most beneficial in case of extreme posterior meniscal rips. Instruments used for repair (such as screws, staples, etc.) are commonly made of bioabsorbable compounds like poly-L lactic acid [15]. These implants are deformable and hence lowering the potential of chondral erosion during weight-bearing [15]. Although arthroscopic techniques aim to lower the risk of neurovascular problems, inadvertent damage can still occur in all the above [15].

Meniscal Reconstruction

The least commonly performed is the meniscus reconstruction surgery, in which attempts are made to replace missing/resected components of the native meniscus with functional ones [14]. The aim of this procedure is to re-establish the functionality of the knee joints and mitigate degenerative processes that would otherwise result from poor knee biomechanics [14]. Reconstruction can be performed with the use of either meniscal scaffolds or via meniscal allograft transplantation (MAT) [14]. MAT involves the transplantation of preserved meniscus allograft. Meniscal scaffold surgery, on the other hand, uses synthetic biodegradable porous structures to fill meniscal defects [14]. The high porosity of the scaffolds allows vascular tissue to grow within them which provides additional reinforcement [14].

Finally, postoperative rehabilitation and gradual return to normal activity are encouraged to optimize outcomes with all of the above surgeries [14].

Cell-Based Tissue Engineering

Tissue engineering (TE) is an upcoming technology that has the potential for use in the treatment of meniscal tears [18]. Regeneration of the meniscus is the primary concept employed, which is met by stimulating the differentiation of cells into tissue that has phenotypical features identical to the native meniscus [18]. This technology can be used not only for the repair of meniscal tears but also for the regeneration of a partial or complete meniscus following a partial, subtotal, or total meniscectomy [18].

The most common cell types used in meniscal TE include meniscal cells, articular cells, and mesenchymal stem cells (MSC) specifically, embryonic, bone marrow, and synovium derived [18]. These cells can proliferate and differentiate into cartilaginous cells with the deposition of extracellular matrix (ECM) found in the native meniscus [18]. As per a study by Marsano et al., MSC led to tissue samples with the greatest resemblance to the human meniscus particularly, with larger depositions of ECM including glycosaminoglycans and collagen types I, II, and IV [18]. Hence, making MSC an attractive cell type for meniscal TE [18]. Additionally, MSCs also exhibit multilineage differentiation and self-renewal capacity, which make them the perfect substrate for regeneration [18]. They can also re-establish joint homeostasis and enhance tissue repair via the secretion of paracrine and anti-inflammatory factors [18]. Furthermore, bone-marrow-derived MSC remains the main cell source of MSC-based TE. Primarily, because they have a higher osteogenic, chondrogenic, and adipogenic potential and because they can be harvested with ease and limited morbidity [18]. Importantly, the bone marrow also encompasses hematopoietic stem cells; which are distinguished from MSC due to the presence of cell surface antigens, including CD45 and CD34 [18]. Moreover, growth factors have also been used in TE to further promote cell proliferation/differentiation and deposition of ECM while inhibiting tissue metalloproteinase and improving vascularization of the engineered tissue [18]. The commonly used growth factors include transforming growth factor β (TGF-β), hepatocyte growth factor (HGF), insulin-like growth factor 1 (IGF-1), fibroblast growth factor 2 (FGF-2), and platelet-derived growth factor (PDGF) [7].

There are two ways of administering MSC and growth factors for the purpose of meniscal TE: (1) intra-articular injections and (2) seeding onto a biomaterial meniscal construct called a scaffold. The main advantage of IA injections is that they can be performed with limited morbidity, can be repeated, and can prevent the systemic diffusion of injected cells [18]. In terms of a scaffold, the most ideal characteristics would be: (i) cell-instructive, allowing proliferation and differentiation of the seeded cells, (ii) architectural mimic of the native meniscus, (iii) resilience to mechanical forces acting on the joint while allowing deposition of ECM, (iv) biocompatibility to prevent an immunogenic reaction, and (v) easily implantable [18]. As per Chiari et al. study, both natural allogeneic and synthetic scaffolds have been used but are singly not sustainable [18]. Calling for the need for a hybrid coupling of the biocompatibility of allogeneic scaffold with the mechanical strength of synthetic scaffold in order to tailor a more sustainable scaffold [18].

As per available literature, preclinical studies on cell sources, scaffolds, and growth factors have shown several limitations along with no clear benefits; thereby making the clinical practice of TE controversial [18]. Advancements in genetics and bioengineering are, therefore, required to solve the clinical challenges [18].

## Conclusions

The menisci were thought to be a functionless structure and were resected completely decades ago. Since the menisci were actually known to play an essential role in the biomechanics of the knee, the orthopedic research on menisci shifted from resection to preservation. Today, a meniscus tear is a very common injury and its incidence is increasing in all ages either due to trauma or osteoarthritis. The recent advances in ongoing research studies and clinical trials contribute to the diagnosis and management of the condition. A thorough investigation of the patient history, physical examination, as well as meniscal tear characteristics, will facilitate a better understanding of the pathogenesis and therapy. The vascular supply of the knee joint plays a major role in the healing and repair of the tissue properly. Therefore, identifying the correct location of the lesion will contribute to an optimal treatment plan and postoperative rehabilitation. Although conservative management is preferred for some patients and has its role in a patient's functional improvement, surgery remains the main treatment for meniscal tears. Despite having the advantage of being minimally invasive, fast recovery, and low complications, partial meniscectomy still leads to osteoarthritis in the long run. Arthroscopic meniscal repair has been popularizing in the last decade and became the procedure of choice for meniscal lesions. Although meniscal repair has shown promising results and a low short-term failure rate, the long-term failure rate is reported to reach up to 30% consistently. Cell-based tissue engineering is a newer therapy with a concept of regeneration of the menisci by stimulating other cells. Studies have limitations and the therapy is yet to be proven to be beneficial in both the short and long run, thus more evidence-based research studies are required. Greater efforts in developing modern imaging and technologies will continue to provide advanced tools to further develop diagnostic and treatment interventions.
